# Interactions of Hydroxyapatite with Proteins and Its Toxicological Effect to Zebrafish Embryos Development

**DOI:** 10.1371/journal.pone.0032818

**Published:** 2012-04-11

**Authors:** Zhen Xu, Ya-Lei Zhang, Cao Song, Ling-Ling Wu, Hong-Wen Gao

**Affiliations:** 1 State Key Laboratory of Pollution Control and Resource Reuse, College of Environmental Science and Engineering, Tongji University, Shanghai, China; 2 Key Laboratory of Yangtze River Environment of Education Ministry of China, Tongji University, Shanghai, China; Northeastern University, United States of America

## Abstract

The increased application of nanomaterials has raised the level of public concern regarding possible toxicities caused by exposure to nanostructures. The interactions of nanosized hydroxyapatite (HA) with cytochrome c and hemoglobin were investigated by zeta-potential, UV-vis, fluorescence and circular dichroism. The experimental results indicated that the interactions were formed via charge attraction and hydrogen bond and obeyed Langmuir adsorption isotherm. The two functional proteins bridged between HA particles to aggregate into the coralloid form, where change of the secondary structure of proteins occurred. From effects of nanosized HA, SiO_2_ and TiO_2_ particles on the zebrafish embryos development, they were adsorbed on the membrane surface confirmed by the electronic scanning microscopy. Nano-HA aggregated into the biggest particles around the membrane protein and then caused a little toxicity to development of zebrafish embryos. The SiO_2_ particles were distributed throughout the outer surface and caused jam of membrane passage, delay of the hatching time and axial malformation. Maybe owing to the oxygen free radical activity, TiO_2_ caused some serious deformity characters in the cardiovascular system.

## Introduction

Recently, the growing industrial usage of nanomaterials brings an urgent need for information on their potential health effects [1], [2], [3], [4], [5], [6], [7]. Due to their distinctive physico-chemical characteristics of nanomaterials including chemical reactivity, their possible toxicity may differ from that of the bulk material of similar chemical nature [8], [9], [10], [11], [12]. Nanomaterial particles are smaller than cells so that they may penetrate various tissue or even cell and then produce a physical damage. For example, oxidative stress caused by nano-sized particles can damage lipids, carbohydrates, proteins and DNA; in particular, lipid peroxidation is considered most dangerous as it alters cell membrane properties [13], [14], [15], [16]. Many epidemiological and experimental studies have indicated that ultrafine particles are closely associated with respiratory and cardiovascular diseases [16]. There is evidence that nanomaterials including nano-hydroxyapatite (nano-HA), nano-SiO_2_ and nano-TiO_2_ can cause inflammation, fibrosis, pulmonary damage and even DNA damage [17]. Nanoparticles eluded the phagocytic pathway and a few were even seen to enter the nuclei through nuclear pores [11].

Functional proteins play an important role in organisms. Cytochrome c (cyt c) can catalyze the hydroxylation and aromatic oxidation, and show peroxidase activity by oxidizing various electron donors [18]. Hemoglobin (hb) is well known for its oxygen carrier function in animal vascular systems. It also aids, both directly and indirectly, in carbon dioxide transport and regulates pH of blood [19]. The interactions of nanomaterials with functional biomolecules such as proteins, enzymes and DNA are regarded as preconditions for their cytotoxicity and organ toxicity. Various proteins have been investigated during recent years, e.g. fibrinogen, human serum albumin and lysine [20], [21], [22], [23]. For example, Larsericsdotter reported electrostatic effects on protein adsorption isotherm using differential scanning calorimetry [24]. Motskin indicated that nano-HA can be toxic. The majority of sequestered nanoparticles and microparticles ended up in the phagocytic pathway and were dissolved over time within lysosomes. Some escaped this pathway and ended up in the cytoplasm and a few even translocated to the nucleus [11]. Nezu and colleagues reported that electrostatic interaction was the main mechanism controlling the adsorption of lysozyme to SiO_2_ [25].

As well known, any substance e.g. chemical can travel through cell membrane and then be carried to nucleus and genes by the carrier proteins in cytoplasm, thus causing inner toxicity. Their interaction with proteins and DNA may help people understand its toxicity-causing mechanism.

The toxicity effect can be reflected from the developmental status of embryos. Zebrafish is a universal model for the study of ontogenetic development, pharmacology, and toxicology. It is a kind of small freshwater fish with a short lifecycle, high fecundity, and especially the transparent trait during early life stage, which makes it easy to visualizing vital dyes, fluorescent markers, antibodies, and riboprobes in live and whole mounted fixed specimens [26]. Zebrafish embryo has been proved to be a good model vertebrate to assess the toxicity of nanoparticles [17], [26], [27]. Ba and colleagues reported that nano-ZnO killed zebrafish embryos (50 and 100 mg/L), retarded the embryo hatching (1–25 mg/L), reduced the body length of larvae, and caused tail malformation after the 96 hpf exposure [28]. Yeo reported that the hatching rate of zebrafish embryos decreased in the nano-silver exposed groups (10 and 20 ppt); furthermore, the hatched flies had an abnormal notochord, weak heart beat, damaged eyes and curved tail. The expression of the Sel N1 gene decreased in the nanosilver exposed groups, and the catalase activities of the exposed groups increased relative to those in the control group [25]. Though nanosized materials cross the membrane pores into cytoplasm causing various toxicities above, they may be adsorbed on the out surface of membrane to cause a physical injury and then influence the normal physiological metabolism of cell.

The objective of this work is to identify the effects of nano-HA on protein conformation and their binding type and compare the toxicological effect of three nanosized materials (HA, SiO_2_ and TiO_2_) on development of zebrafish embryos, including mortality, hatching rate and so on.

## Results

### Aggregation of proteins on nano-HA particles

**Figure 1:**
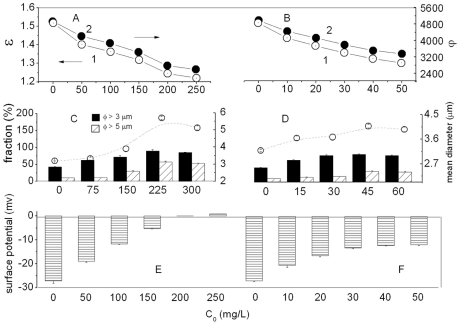
Variation of various factor*s* measured in the HA-proteins liquids. Change of *ε* (1) and *ϕ* (2) of 500 mg/L nano-HA in the presence of cyt c (A) and hb (B). The size (>3 µm and >5 µm) fraction (C and D) and surface potential (E and F) of the suspending particles in the presence of cyt c (C, E) or hb (D, F) at pH 7.4 in 0.15 M NaCl.

In order to evaluate change of the HA particles in the presence of cyt c and hb, the light absorption of liquids (*A*) was measured from 450 to 600 nm (Fig. S1 A and B). Plots lg*A* vs. lg*λ* was regressed (Fig. S1 C and D). From the relation 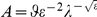 [29] (*λ*- wavelength, *ϕ*- number factor, proportional to particle number and *ε* - size factor, inversely proportional to particle size). HA-particles increased in size with increase concentrations of cyt c from 0 to 300 mg/L and hb from 0 to 60 mg/L. For example, only 43 and 10% of the HA particles were more than 3 and 5 µm in the absence of protein (Fig. 1 C), but these more than 90 and 57% appeared in 300 mg/L cyt c. The initial mean size 3.19 µm increased to 1.6-fold when the cyt c exceeded 250 mg/L. With the same method, 60 mg/L hb also saturated the HA particle surface (Fig. 1 D).

**Figure 2:**
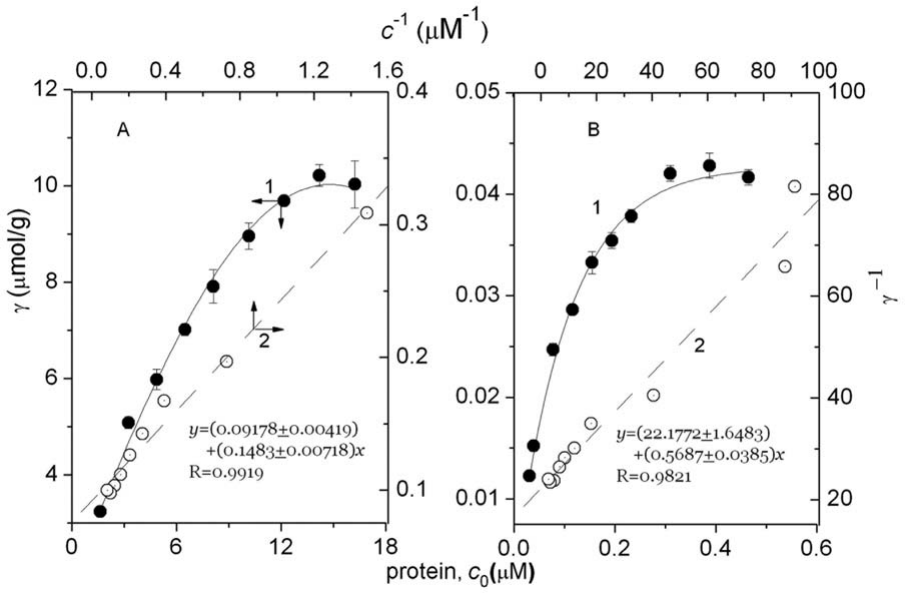
Variation of γ of proteins in the presence of nano-HA. 1: γ of cyt c (a) and hb (b) in the presence of nano-HA. a- the suspensions containing 500 mg/L nano-HA and 10 to 200 mg/L cyt c and b- 2500 mg/L nano-HA and 20 to 300 mg/L hb. All of the liquids were at pH 7.4 in 0.15 M NaCl. 2: Plots γ^−1^ vs. *c_L_* ^−1^ of the above liquids.

The surface potential of HA was determined as illustrated in Fig. 1 E and F. From the column height in Fig. 1 E, the ζ-potential of the HA-cyt c aggregates decreased with increase of cyt c. For example, ζ-potential decreased from −27.12 to +0.83 mv in 300 mg/L cyt c. The ζ-potential of HA decreased gradually with increase of hb (Fig. 1 F). According to the recommended procedure, the free proteins existed in the HA-proteins liquids were determined from its supernatants and its mole number binding to proteins calculated. Their relationship was given in Fig. 2. From curves 1, the number (γ) of proteins bound to HA particles increased with increasing cyt c and hb concentration. All γ values approached constant maxima when protein (*c*_0_) was more than 1.75 g/L in cyt c solution and 2.50 g/L in hb solution. The Langmuir isotherm equation: 
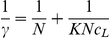
 (*N* - the saturation number of protein, *c*_L_ - the equilibrium molarity of protein and *K* - the adsorption constant, M^−1^) [30] was used to fit the data. Plots γ^−1^ vs. *c*_L_^−1^ showed a good linearity (Fig. 2).

The fluorescence spectra of the HA-protein suspensions were measured. The intensity decreased sharply with increase of HA (Fig. S3 A and C), but *c*_L_ of the free cyt c and hb decreased slowly in the supernatant. (Fig. S3 B and D). For example, the fluorescence of free cyt c changed by less than 10% in 50 mg/L HA but that of the suspension decreased by 75%. When HA is more than 700 mg/L, over 90% fluorescence of free cyt c was scattered and over 60% that of free hb.

### Identification of the binding subdomain

CD is often used to characterize the secondary structure of a protein with β-pleated sheet, β-turn, α-helix and random coil. The HA particles have a dramatic effect on the secondary conformation of cyt c and hb (Fig. S4). Being similar to the adsorption of lysozyme on TiO_2_ nanoparticles, the α-helix and β-turn of cyt c decreased obviously, but the β-pleated sheet increased with increasing HA. For example, the α-helix of cyt c decreased from 33.7% in absence of HA to 24.0% in 200 mg/L HA and the β-pleated sheet increased from 0 to 20.8% (Table 1). By contrast, the α-helix and β-turn fractions of hb increased with increasing HA particle numbers but the β-pleated sheet increased from 0 to 20.8% (Table 1). By contrast, the α-helix and β-turn fractions of hb increased with increasing of that of HA particles but the β-pleated sheet decreased (Fig. S4 B). For example, the α-helix of hb increased from 42.6% in absence of HA to 52.8% and in 200 mg/L HA and its β-pleated sheet decreased from 19.6% to 0 (Table 1), being similar to organic compound binding to lysozyme [31].

**Table 1:** Change of the secondary conformation factors of cyt c and hb in the presence of nano-HA particles.

nano-HA added into		α-helix	β-pleated sheet	β-turn	Random coli
cyt c	A0*	33.70±1.85	0.00±0	31.60±1.28	34.70±0.79
	A1	24.00±0.45	20.80±0.56	23.60±1.39	31.50±1.46
hb	B0	42.60±0.47	19.60±0.74	15.70±0.75	22.00±0.42
	B1	54.80±1.86	0.00±0	26.40±0.92	18.80±0.43

* : A0 and B0- the absence of nano-HA; A1 and B1- 200 mg/L nano-HA added in cyt c and hb solutions. All are three replicated determinations.

### Effects of pH, ionic strength and temperature

The effects of pH, electrolyte and temperature on the interactions of the two proteins with HA were determined according to the recommended procedures (Fig. S5). The ζ-potential of HA particles increased at pH>8 (Fig. S6). Being similar to the interactions of cyt c, dnase II and hb with nano-SiO_2_ [32], γ increased as the pH rose from 4.5 to 8.4 (Fig. S5 A and D). Although the HA particle ζ-potential increased at pH above 8 (Fig. S6), the Lys and Arg residues were less positively charged, so the charge attraction between protein and HA particle weakened and γ decreased. γ decreased as the temperature rose from 25 to 55°C (Fig. S5 B and E) and decreased sharply with increasing electrolyte (Fig. S5 C and F).

### Effect of HA particles on the activity of cyt c

**Figure 3:**
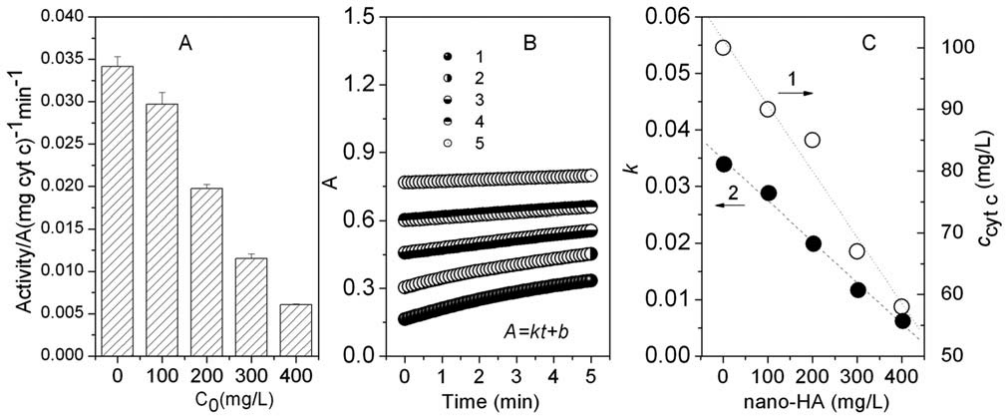
Effect of nano-HA on the cyt c activity. A: Changes in cyt c peroxidase activity, where the suspensions contained 100 mg/L cyt c and 0 to 400 mg/L nano-HA particles at pH 7.4 in 0.15 M NaCl. B: Effect of time on absorbance of the nano-HA-cyt c suspensions initially containing 0.100 g/L cyt c, 0 to 400 mg/L nano-HA (curve 1 to 5), 25 mM ABTS, 120 mM hydrogen peroxide, and 0.15 M NaCl at pH 7.4, all measured against the reagent blank. C: Effect of nano-HA on the activity rate constant (*k* = d*A*/d*t*) (1) and free cyt c (*c*_cyt c_) (2).

The protein conformation always corresponds to its function, so structural change may alter normal physiological activity [33]. The function of cyt c is to transport electrons from cyt c_1_ to cytochrome oxidase, a shuttle connecting two respiratory chain energy transducers [34]. The activity of cyt c was determined in HA media in Fig. 3, where cyt c inactivated approximately 68% in 300 mg/L HA.

The dynamic change of cyt c activity in HA (Fig. 3 B) corresponded to the linear equation: 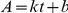 where *t* is the reaction time (min), *k* i.e. d*A*/d*t* the activity rate constant and *b* the regression constant.

### Effects of three nanomaterials on development of zebrafish embryos and larvae

**Figure 4:**
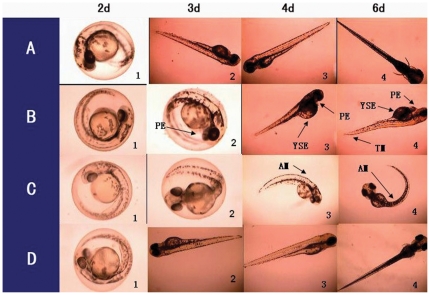
SEM images of Zebrafish embryos. Embryos exposed for 8 h in distilled water (A), 400 mg/L nano-TiO_2_ (B), 400 mg/L nano-SiO_2_ (C) and 400 mg/L nano-HA (D). The freeze-drying of embryos was carried out at −55°C for 12 h.

In the 200 mg/L nano-TiO_2_ exposure group (Fig. S7 B), none of the embryos hatched out at 3 dpf, and some hatched out at 3.5 dpf (Fig. S7 B) with obvious pericardial edema (PE) and yolk sac edema (YSE) (Fig. 4 B-3, 4). In the 400 mg/L nano-TiO_2_ exposure group, 30% of embryos and larvae showed PE and YSE (Fig. S7 E, Fig. S7 H). However, none of embryos and larvae had YSE in the HA exposure group. None of the embryos died before hatching (3 dpf) (Fig. S7 J and L). The mortality exhibited an obvious toxicity in >100 mg/L TiO_2_. All embryos and larvae died in 400 mg/L TiO_2_ for 5 days exposure, while only 30% died in 400 mg/L HA and SiO_2_ (Fig. S7 J, K and L).

## Discussion

From curves 1 in Fig. 1 A, B, the decrease of ε indicated that larger particles were formed in the presence of proteins. It may be attributed to cyt c and hb binding to the particle surface. From curves 2, decrease of *ϕ* with increasing proteins exhibited the aggregation of particles intensified in the presence of proteins. These were confirmed by their SEM images (Fig. S2). The HA-only particles readily formed irregular self-aggregates, but they adhered each other into coralloid in the presences of cyt c and hb. Proteins therefore induced the aggregation of HA particles.

From change of the HA surface potential (Fig. 1 E and F) the HA particle surface carried a number of negative charges. As is well known, cyt c consists of 104 of amino acid residues, 24 of which are basic (Lys, His and Arg) and 12 acidic (Asp and Glu). The isoelectric point of cyt c is at 10.6 [35] and His residue is positive at pH 7.4. Therefore, the positive-negative charge attraction induced cyt c binding to HA, being similar to the interaction of lysozyme with TiO_2_ and that of SiO_2_ with cyt c, dnase II and hb. The side groups of 21 Lys residues including 5, 7, 8, 13, 22, 25, 27, 39, 53, 55, 60, 72, 73, 79, 86, 87, 88, 99 and 100 and Arg38 and 91 might bind directly to the HA surface. The isoelectric point of hb at 6.8 [36] and it consists of 4 peptide chains with 572 amino acid residues, 62 of which are charged positively, i.e. Lys7, 11, 16, 40, 56, 61, 68, 90, 99, 127 and 139, and Arg31, 92 and 141 on chain a; Lys7, 16, 18, 58, 65, 75, 81, 94, 103, 119 and 131, and Arg29, 39, 115 and 143 in chain b; Lys7, 11, 16, 40, 56, 61, 68, 90, 99, 127 and 139, and Arg31, 92 and 141 in chain c; and Lys7, 16, 18, 58, 60, 64, 65, 75, 81, 94, 103, 119 and 131, and Arg29, 39, 115 and 143 in chain d. In contrast to cyt c, hb carries a net negative charge at pH 7.4. The ζ-potential of the HA-hb aggregate therefore approached a constant value of −11 mv in any hb concentration (Fig. 1 F).

From curves in Fig. 2, the interactions obeyed the monolayer adsorption. From the intercepts of lines 2 in Fig. 2, *N* values of cyt c and hb were calculated to be 10.2±0.2 and 0.043±0.001 µmol per gram of HA. From the slopes, their *K*s were calculated to be 3.90×10^7^ and 4.73×10^5^ M^−1^. Therefore, proteins firmly bound to HA particles. *N* decreased with increasing peptide chain length. It indicates that the binding depends on both the length and the steric effect of the peptide chain. *K* increased with increasing number of Lys and Arg residues. In addition to the positively and negatively charged residues, proteins contain polar residues e.g. Thr, Tyr, Asn, Gln, Cys and Ser. In fact, hydrogen bond formed between HA and these polar groups in the N-H···O and O-H···O form, when the positively charged side groups bound to HA. For example, the polar residues of cyt c, e.g. Thr89, 63, 58, 49, 47 and 28, Tyr74, Asn103, Gln12, 16 and 42, and Cys14 and 17 locating around the residues positively charged, also bound to HA via hydrogen bond. The *N* values of the two proteins (Fig. 2) were much higher than that of OVA [37]. As a result, the electrostatic attraction was the main interaction inducing force [37]. Thus, the proteins bound to HA particles by a combination of charge attraction and hydrogen bond.

**Figure 5:**
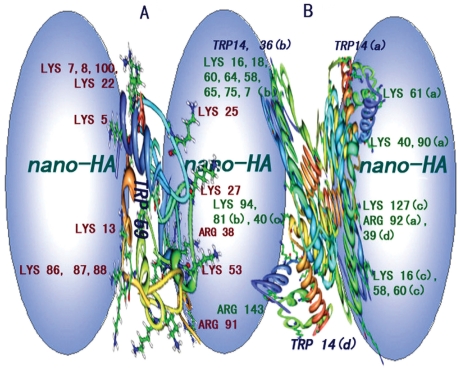
Cartoon illustration for proteins binding to nano-HA. A: cyt c and B: hb. Positive-negative charge attraction pulled the peptide chain binding on the particle surface and N-H···O and O-H···O hydrogen bonds formed. Twisting and transmutation of the peptide chain is illustrated intuitively.

The light scattering of HA particles was obvious so that the fluorescence quenching of the liquids occurred obviously. An additional reason is that the side groups of tryptophan residues: Trp59 in cyt c and A-Trp14, B-Trp36, C-Trp14, D-Trp14 and D-Trp36 in hb (Fig. 5) may directly bind to HA particles via the N-H···O and O-H···O hydrogen bond owing to twist and deformation of cyt c and hb. A red shift of the emission peak appeared (Fig. S3 A–D). It is attributed to a strong light-scattering of HA particles at short wavelength. Moreover, the presence of HA had not affected the conformation of protein freely existed in the liquids.

The α-helix area of cyt c bound directly to the HA particles and the press and pull interaction broke the original hydrogen bonds on α-helix, changing the spiral peptide chain into a sheet-like conformation (Fig. 5A). By contrast, the β-pleated sheet area of hb bound directly to the HA particles and the press and pull interactions twisted the sheets (Fig. 5 B). Thus, a helix-like structure may be formed. Such a conformation change may alter the microenvironment around Trp residue and then caused the fluorescence quenching (Fig. S3 B).

In pH>8 media (Fig. S6), Lys and Arg residues were less positively charged, so the charge attraction between protein and HA particle weakened, i.e. γ decreased. From γ decreasing with rise of temperature, the protein conformation may be expanded at a higher temperature. It is different from the adsorption of lysozyme on nano-TiO_2_ but similar to the interactions of proteins with SiO_2_. The γ decreasing sharply with increasing ionic strength reflected that electrostatic attraction is the main interaction force. The double electric layer on HA particles adsorbed Na^+^, so the positively charged side groups of proteins are repelled. The induction, orientation and dispersion forces between a protein and HA got stronger in a high salt medium owing to polarization, which is different from the interaction between HSA and nano-TiO_2_.

From Fig. 3 C, Plots *k* vs. *c*_HA_ was linear and the activity rate constant was inversely proportional to HA content. This is attributable to the fact that positively charged His residues bound to the HA particle surface. The active site was covered and therefore inactivated. The cyt c binding to HA particles had no activity but free cyt c remained active. HA particles inactivated cyt c but caused no synergistic effect, in contrast to other reports.

In an organism, any substance including nanosized materials may enter cytoplasm through cell membrane then to cause toxicity. However, it was inevitable to interact with the membrane phospholipid and membrane protein during its transmembrane. Certainly, the transmembrane impedance effect (TMIE) [38] excludes any unwanted substance, especially nanosized ones owing to a much greater steric effect. From the SEM images (Fig. 4), the SiO_2_ particles were distributed throughout the outer surface. HA and TiO_2_ aggregated together into the bigger particles around the membrane protein when exposed in zebrafish embryos. Thus, the membrane transporter protein conformation may be altered so that the normal physiological metabolism of embryos may be affected.

**Figure 6:**
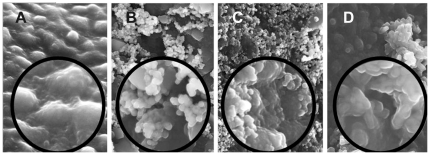
Toxicity characteristics of Zebrafish embryos and larvae. The normal developing embryos and larvae exposed in the reconstituted water (A), 400 mg/L nano-TiO_2_ (B), 400 mg/L nano-SiO_2_ (C) and 400 mg/L nano-HA (D). AM- axial malformation, E- edema, P- pericardial, PE- pericardial edema, YS- yolk sac and YSE- yolk sac edema.

**Figure 7:**
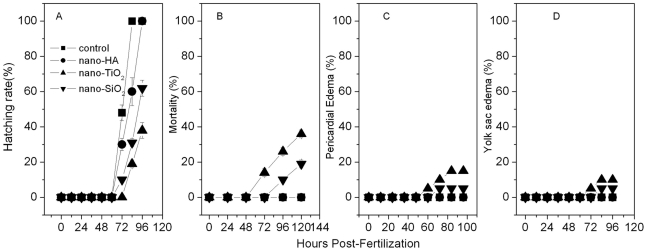
Toxicity-causing rate of Zebrafish embryos and larvae. A: Hatching rate, B: Mortality, C: PE and D: YSE. Exposed in 100 mg/L nano-HA, 100 mg/L nano-TiO_2_ and 100 mg/L nano-SiO_2_.

From the photographs taken during zebrafish embryo development (Fig. 6), most of the embryos hatched out at 3 dpf in the control group (Fig. S7 A, B and C, Fig. 7 A). In the 200 mg/L nano-TiO_2_ exposure group (Fig. S7 B), none of the embryos hatched out at 3 dpf, and some hatched out at 3.5 dpf (Fig. S7 B) with obviously PE and YSE (Fig. 6 B-3, 4). Nano-TiO_2_ went through the chorion easily and transported to the developing embryo cell. This disrupted oxidative phosphorylation [39] and reduced myocardial contractility to make the ventricular dysfunction. Hence serious PE and YSE were found in the exposed embryos (Fig. 6 B-2) and larvae (Fig. 6 B-3, 4). In the 400 mg/L nano-TiO_2_ exposure group, 30% of embryos and larvae had PE and YSE (Fig. S7 E, Fig S7 H). This is similar to the effects of Cu_x_TiO_y_ nanometer particles on biological toxicity during zebrafish embryogenesis [40]. However, none of embryos and larvae had YSE in the HA group. Nano-SiO_2_ mainly adsorbed on the out surface of chorion (Fig. 4 C), which might affect the nutrient absorption and vitamin synthesis to cause the axial malformation (AM) to the exposed embryos and larvae (Fig. 6 C). In nano-HA exposure groups, HA aggregated itself into the biggest particles around the membrane protein (Fig. 4 D), so HA had a little toxicity to the development of zebrafish (Fig. 6 D). However, this is different from the effect of multisized gold and silver manoparticles on zebrafish embryos [27]. From the mortality of the embryos exposed to nano-HA, TiO_2_ and SiO_2_, none of the embryos died before hatching (3 dpf) in the HA and SiO_2_ exposure groups (Fig. S7 J and L). The mortality data showed obvious toxicity in more than 100 mg/L nano-TiO_2_. All embryos and larvae died in 400 mg/L nano-TiO_2_ for 5 days exposure, while only 30% died in 400 mg/L HA and SiO_2_ for 5 days (Fig. 7). It is due to the oxygen free radical activity formed by nano-TiO_2_ [41].

In conclusion, the adsorption of two functional proteins to nano-HA obeyed the Langmuir isotherm model. Charge attraction induced protein binding to nano-HA particles by electrostatic attraction and hydrogen bond. The acidity and ion strength of the media markedly affected the interaction. The protein binding area and binding site were estimated by ζ-potential, fluorescence and CD and then the binding model was inferred. Nano-HA particles changed the secondary conformation of proteins by twisting and pulling the peptide chains. Nano-HA particles inactivated cyt c activity but caused no synergistic effect. By comparing three nanosized materials, e.g. HA, TiO_2_ and SiO_2_, they were adsorbed on the cell membrane. The SiO_2_ particles were distributed throughout the outer surface, which caused the jam of membrane passage, delay of the hatching time and axial malformation. HA aggregated into the biggest particles around the membrane protein and then caused a little toxicity to development of zebrafish embryos. Owing to the oxygen free radical activity, TiO_2_ causes some serious deformity characters in the cardiovascular system. The method established in this work could be used to understand the interaction of nanomaterials with proteins and their influence on the development of zebrafish.

## Materials and Methods

### Instruments and materials

The absorption spectra of all suspensions/solutions were recorded with a Model Lambda-25 spectrometer (Perkin-Elmer, USA) equipped with a thermostatic cell holder to link with a Model TS-030 water-circulated thermostatic oven (Yiheng Sci. Technol. Shanghai, China). A Model J-715 CD Spectropolarimeter (Jasco Instrum., Japan) was used to measure protein conformations. Fluorescence spectra were recorded with a Model F-4500 Fluorescence Spectrophotometer (Hitachi, Japan). ζ-potential was measured with a Model Zetasizer nano ζ-Potential Analyzer (Malvern Instruments, UK). A Model LS230 Particle Size Analyzer (Beckman Coulter, USA) with a Model LFC-101 Laser Channel (Ankersmid Ltd., Holland) was used to measure the distribution of the aggregates. A freeze-drying instrument (Model K750X, Jintan Etong Electrons, China) was used to prepare the lyophilized embryos. A scanning electron microscopy (SEM) (Model S-4800, Hitachi Inc., Japan) was used to measure morphology of particles and chorion. An ultrasonic cleaning device (Model SK3300H, Shanghai Ultrasonic Cleaning Instruments, China) was used to accelerate the dispersion of nanomaterials. An inverted microscope (Model TE2000-U, Nikon Inc., Japan) with the charge-coupled device (CCD) (Evolution™ MP, Media Cybernetics, Japan) and digital photomicrography computer software (Image-Pro *Plus* 6.0) was used to observe the toxicity characteristics of embryos and larvae.

Nano-HA (5000 mg/L) (average particle size 20 nm, Nanjing Emperor Nano Material Co., Ltd. China) was used without further modification. It was suspended in deionized water and mixed ultrasonically for 10 min before use. Cyt c from horse heart (2000 mg/L) (Sigma, USA) was freshly prepared. Hemoglobin from bovine blood (2000 mg/L) (Shanghai Chemical Reagents, China Med. Group) was dissolved in deionized water and stored at less than 4°C. Britton-Robinson (B-R) buffers, from pH 4.0 to 8.4, were prepared to adjust the acidities of solutions, and the ionic strengths were adjusted with 1.5 M NaCl. The other reagents were purchased from China Med. Group. Methyl green was obtained from Shanghai Kayon Biological Technology (China).

### Photometric determination of nano-HA-protein interaction

Proteins and nano-HA were mixed in 10-ml calibrated flasks containing 10 to 200 mg/L cyt c and 500 mg/L nano-HA particles or 20 to 300 mg/L hb and 2500 mg/L nano-HA particles; 2.0 ml of pH 7.4 B-R buffer and 1.0 ml of 1.5 M NaCl were added. Each suspension was diluted to 10.0 ml with deionized water and mixed thoroughly. The calibrated flasks were incubated in a water bath at 37°C with magnetic stirring (500 rpm). After 12 h, the solids with adsorbed proteins were separated by centrifugation at 10,000 rpm for 10 min. The absorbances of the supernatants containing cyt c and hb were measured at 405 nm by UV-VIS spectrophotometry. A reagent blank without cyt c or hb was prepared simultaneously by the same procedures.

Using these procedures, a series of suspensions were prepared initially containing 0 to 250 mg/L cyt c, 500 mg/L nano-HA, 2.0 ml of pH 7.4 B-R buffer and 1.0 ml of 1.5 M NaCl. And another series of suspensions were prepared initially containing 0 to 50 mg/L hb, 500 mg/L of nano-HA, 2.0 ml of pH 7.4 B-R buffer and 1.0 ml of 1.5 M NaCl. Each suspension was diluted to 10.0 ml with deionized water and mixed thoroughly. The calibrated flasks were incubated in a water bath for 12 h at 37°C with magnetic stirring (500 rpm). After 12 h, the absorption spectra of the suspensions were measured between 450 and 600 nm against water.

### Particle size and ζ-potential measurement

The same procedures were used to prepare two series of suspensions initially containing (**a**) 0 to 300 mg/L cyt c and 500 mg/L nano-HA particles, 0 to 60 mg/L hb and 500 mg/L nano-HA particles, and (**b**) 0 to 250 mg/L cyt c and 500 mg/L nano-HA, 0 to 50 mg/L hb and 500 mg/L nano-HA, all containing 2.0 ml of pH 7.4 B-R buffer and 1.0 ml of 1.5 M NaCl. Each suspension was diluted to 10.0 ml with deionized water and mixed thoroughly. The calibrated flasks were incubated in a water bath for 24 h at 37°C with magnetic stirring at 500 rpm. The size distribution of the particles in suspension (**a**) was measured using a particle size analyzer, and ζ-potential of suspension (**b**) was measured with the ζ-potential analyzer.

### Fluorescence measurement

The following suspensions were prepared in 10-ml calibrated flasks: 200 mg/L cyt c, 0 to 700 mg/L nano-HA particles, 250 mg/L hb and 0 to 900 mg/L nano-HA particles. All the suspensions contained 2.0 ml of pH 7.4 B-R buffer and 1.0 ml of 1.5 M NaCl. They were diluted to 10.0 ml with deionized water and mixed thoroughly. After 10 min, the fluorescence spectrum of each suspension was measured with excitation wavelength 280 nm and emission wavelength between 300 and 375 nm (both 5 nm slit widths). The solids with adsorbed proteins were separated by the aforementioned method and the fluorescence spectra of the supernatants were obtained. Simultaneously, a reagent blank without proteins was performed by the same procedure.

### CD measurement

CD spectra were recorded over the range 190 to 250 nm at 37°C using a spectropolarimeter. 100 mg/L cyt c and 70 mg/L hb was mixed with 200 mg/L nano-HA particles respectively. All the suspensions contained 1 ml of pH 7.4 B-R buffer and each was diluted to 10 ml. The suspensions were incubated in a water bath at 37°C with magnetic stirring at 500 rpm. After 24 h, CD spectra were obtained using a 1-mm light path cell. The mean residue ellipticity (MRE) was calculated and corrected with a blank without protein. Three replicate measurements were made. The percent fraction of α-helix, β-pleated sheet, β-turn and random coil were calculated.

### Peroxidase activity of cytochrome c

The peroxidase activity of cyt c was measured using the chromogenic substrate ABTS at pH 7.4 [42]. Each 10-ml calibrated flask contained 0.100 g/L cyt c, 2.0 ml of pH 7.4 B-R buffer, 1.0 ml of 1.5 M NaCl and 0 to 400 mg/L nano-HA particles. These suspensions were put into a water bath and incubated at 37°C for 24 h with magnetic stirring (500 rpm). Free cyt c and cyt c-HA were prepared, and then a fresh ABTS solution was added. Hydrogen peroxide was added to give a final mixture (25 mM ABTS, 120 mM hydrogen peroxide) for incubating native free cyt c and cyt c adsorbed on nano-HA. After the H_2_O_2_ was added, the samples were immediately measured at 415 nm for 5 min. The initial reaction rate and the peroxidase activity were normalized against the cyt c concentration.

### Cultivation, collection and exposure of embryos

The parental zebrafish were kept in a 25-L tank with the following control settings: 250 mg/L hardness, pH 7.5±0.5, 10.5±0.5 mg/L dissolved oxygen. The photoperiod was adjusted to 14 h /10 h of the light/dark cycle at 26±1°C. They were fed regularly with frozen red mosquito larvae from an uncontaminated source. Before testing, several spawning boxes (12×20×12 cm) each containing a mesh (3–4 mm gap) were placed in a tank with 6 male and 3 female fish in each box. Spawning and fertilization took place within 30 min under the light illumination. The fertilized eggs were collected and rinsed with reconstituted water, which had been ventilated closed to 100% oxygen saturation. The normally developing embryos were chosen under an inverted microscope. For valid experiments, fertilized eggs were obtained only from spawns with a fertilization rate higher than 90%. Two hours post fertilization (hpf) embryos and two hours post hatching (hph) larvae were used for exposure.

### Toxicological test of nanomaterials

The embryos were exposed in nano-TiO_2_, nano-SiO_2_ and nano-HA media. 25 ml glass petri dish was used as test chambers for the toxicity bioassays. Ten embryos were exposed in 20.0 ml of liquids and incubated at 26±0.5°C at 14 h light / 10 h dark photoperiod, where every nanomaterial was between 50 and 400 mg/L. The control sample was prepared with the reconstituted water instead of nanomaterial. Photographs of the toxicity characteristics of embryos and larvae were obtained at 2 to 6 dpf with an inverse microscope and the images were compared. The death was defined by cessation of heart beat or coagulation of the embryos and the mortality of the embryos and larvae were calculated. In the tests, dead embryos and larvae were removed from the petri dish in time. Each test was replicated three times consecutively. In addition, in order to observe effects of three nano-sized materials on the structure and shape of the cell membrane surface, the embryos exposed in three nanosized materials. After incubation, the supernatant was removed, and the embryos were freeze-dried for 12 h at −55°C. The SEM morphology of the lyophilized embryos was observed using SEM, and photographs were captured. After incubation for 12 h, each supernatant was removed, and the embryos were incubated with 10 lM DiO for 10 min. The morphology of the exposed embryos was observed using an inverted microscope. Photographs were captured and differences were observed and noted.

## Supporting Information

Figure S1 **Absorption spectra of the HA-cyt c suspensions.** A: containing 500 mg/L HA and cyt c from 0 to 250 mg/L (1 to 6) at pH 7.4 in 0.15 M NaCl and B: 500 mg/L HA and hb from 0 to 50 mg/L. C and D: Plots lg*A* vs. lgλ of the above suspensions.
(TIF)

Figure S2 **SEM images of HA and its protein aggregates.** A: HA, B: the HA– cyt c aggregate and C: the HA –hb aggregate.
(TIF)

Figure S3 **Fluorescence spectra of cyt c and hb in the presence of HA.** A: Fluorescence spectra of HA-cyt c suspensions containing 200 mg/L cyt c and 0, 50, 75, 100, 200, 300, 400, 500, 600 and 700 mg/L HA (1 to 10); B: the same as A but containing 250 mg/L hb and 0, 100, 200, 300, 400, 500, 600, 700, 800 and 900 mg/L HA (1 to 10). All in 0.15 M NaCl at pH 7.4 were measured against the reagent blank without protein. C and D: spectra of the liquids' supernatants without HA.
(TIF)

Figure S4 **CD spectra of cyt c and hb in the presence of HA.** A: suspensions containing 100 mg/L cyt c and 0 (1) and 200 mg/L (2) HA at pH 7.4 and B: ones containing 70 mg/L hb and 0 (1) and 200 mg/L (2) HA at pH 7.4.
(TIF)

Figure S5 **Effects of pH, temperature and electrolyte on γ.** pH (A, D), temperature (B, E) and electrolyte (C, F). ○: the cyt c (80 mg/L) - HA (500 mg/L) liquids and ??? : the hb (100 mg/L) - HA (2500 mg/L) liquids.
(TIF)

Figure S6 **Effect of pH on surface potential of HA.** Variation of the ζ - potential of HA with pH.
(TIF)

Figure S7 **Effects of nanomaterials on hatching rate, pericardial edema, yolk sac edema and mortality.** The hatching rate (A to C), ericardial edema (D to F), yolk sac edema (G to I) and mortality (J to L) of zebrafish embryos and larvae exposed in various nano-HA, TiO_2_ and SiO_2_ suspensions.
(TIF)
