# Supplementary figures and images for: Interactions of Hydroxyapatite with Proteins and Its Toxicological Effect to Zebrafish Embryos Development
Source: PLoS One. 2012 Apr 11;7(4):e32818. doi: 10.1371/journal.pone.0032818 (PMC3324474; doi:10.1371/journal.pone.0032818)

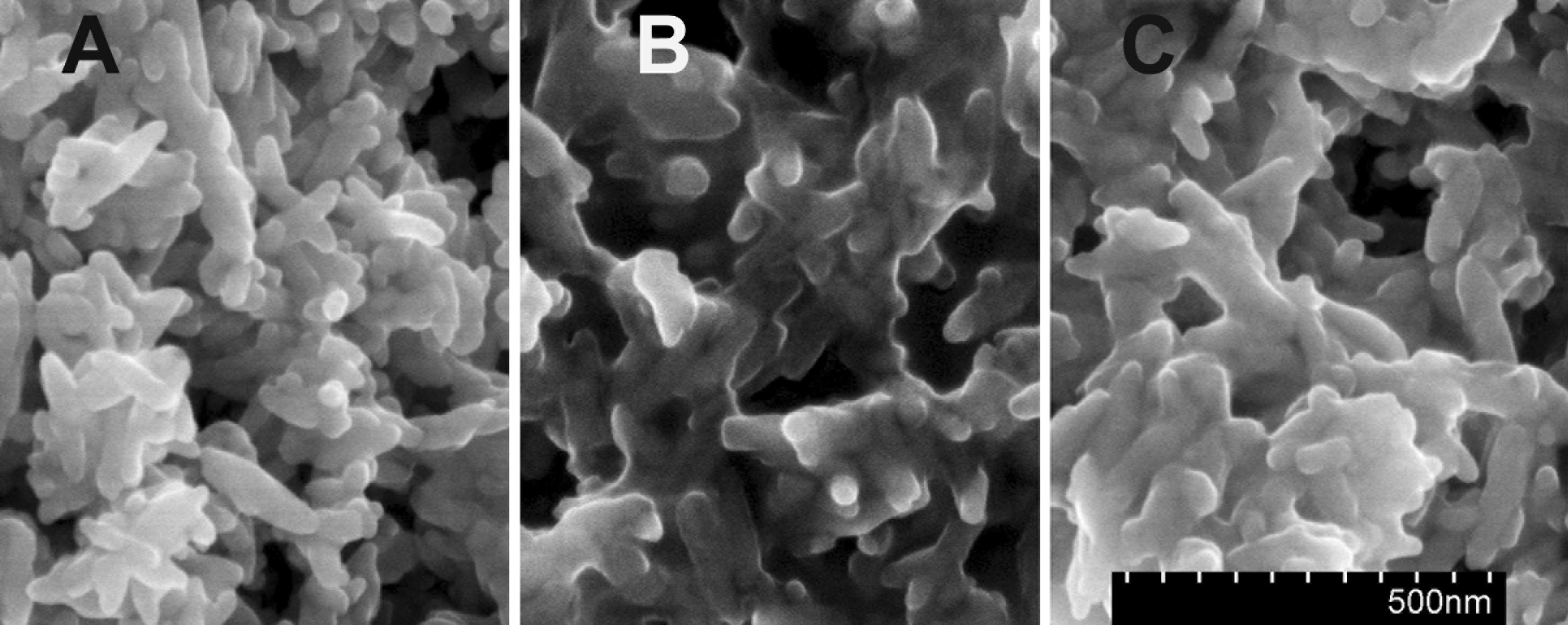

Supplement: Figure S2 — SEM images of HA and its protein aggregates. A: HA, B: the HA– cyt c aggregate and C: the HA –hb aggregate. (TIF) [file pone.0032818.s002.tif]

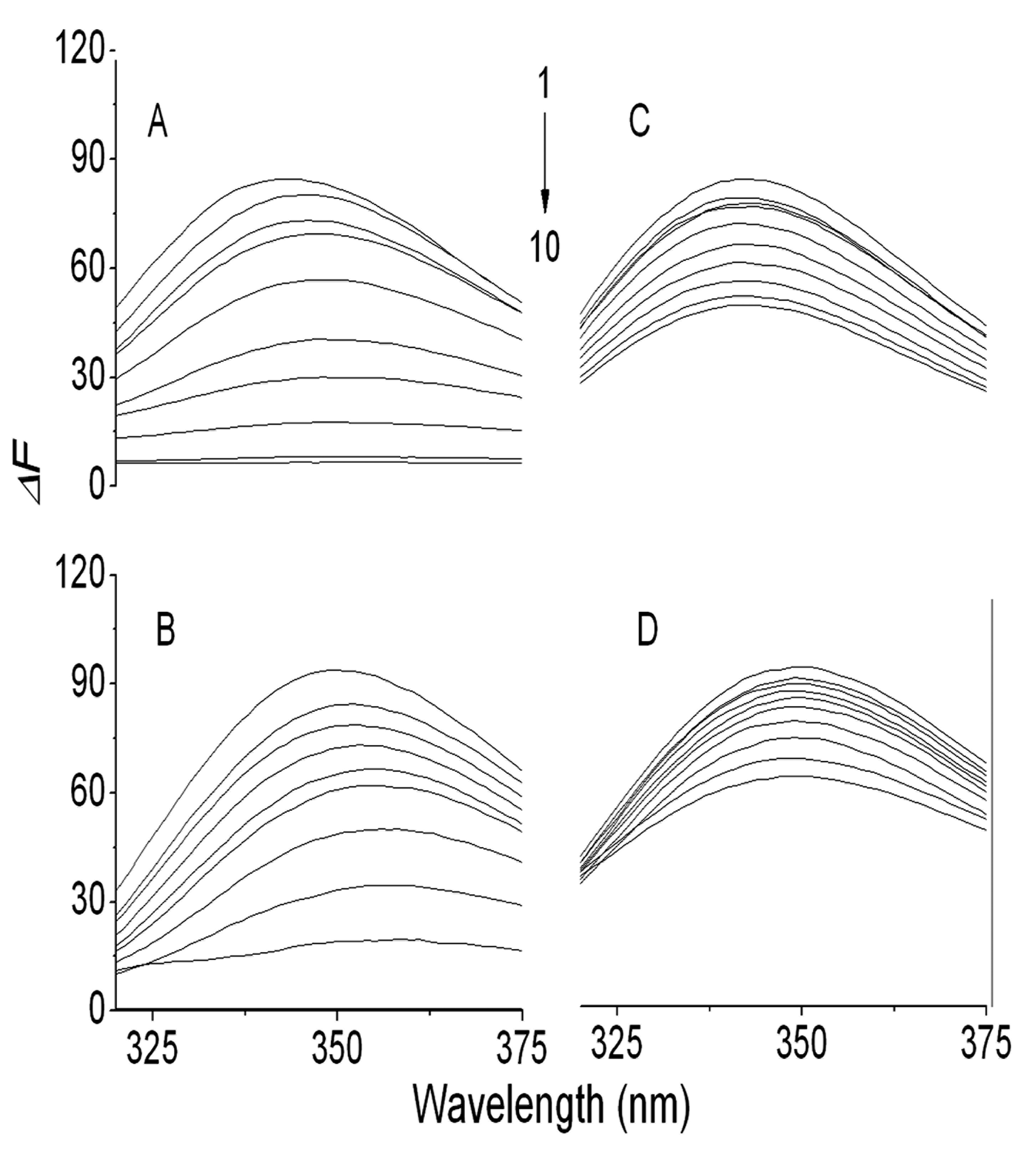

Supplement: Figure S3 — Fluorescence spectra of cyt c and hb in the presence of HA. A: Fluorescence spectra of HA-cyt c suspensions containing 200 mg/L cyt c and 0, 50, 75, 100, 200, 300, 400, 500, 600 and 700 mg/L HA (1 to 10); B: the same as A but containing 250 mg/L hb and 0, 100, 200, 300, 400, 500, 600, 700, 800 and 900 mg/L HA (1 to 10). All in 0.15 M NaCl at pH 7.4 were measured against the reagent blank without protein. C and D: spectra of the liquids' supernatants without HA. (TIF) [file pone.0032818.s003.tif]

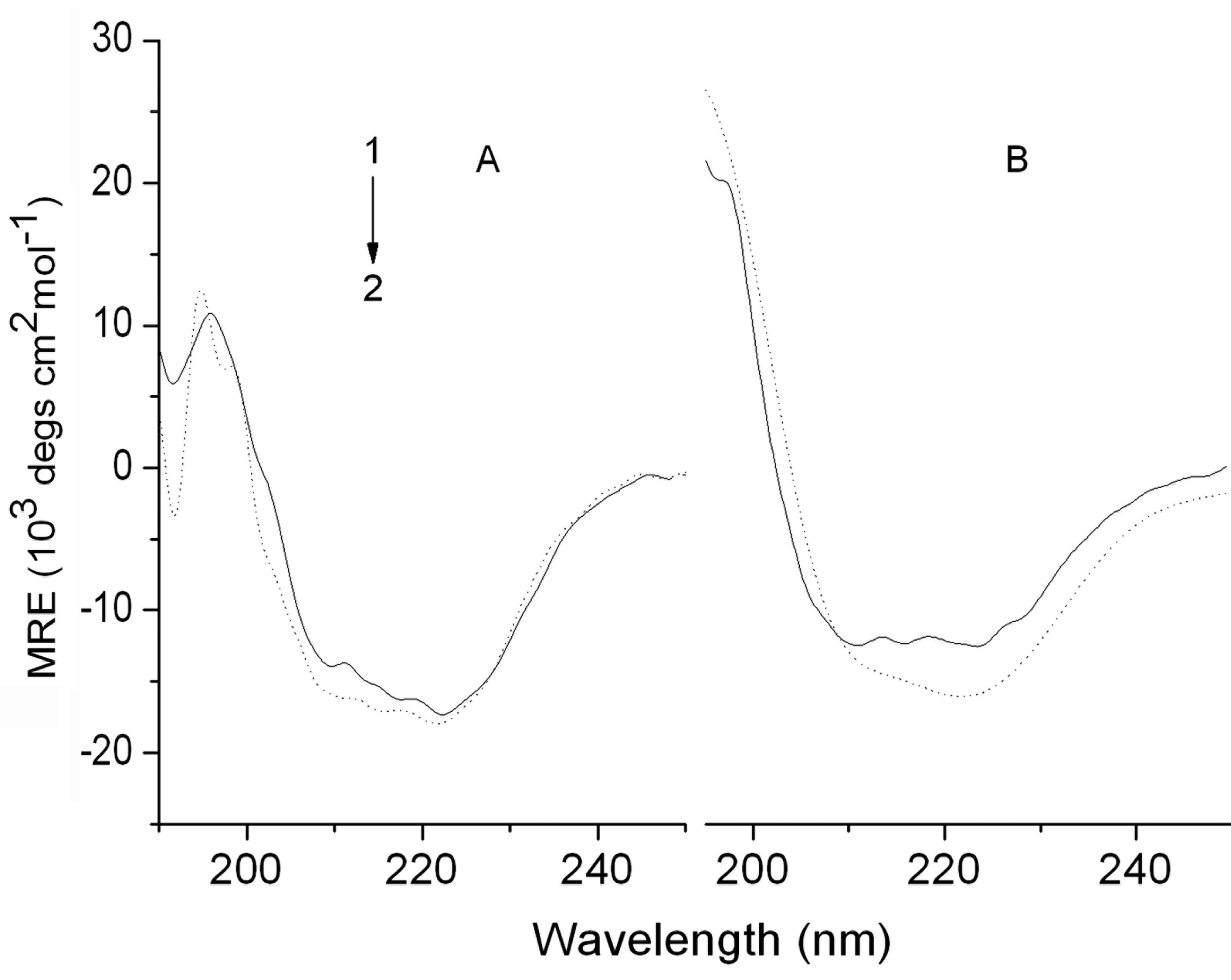

Supplement: Figure S4 — CD spectra of cyt c and hb in the presence of HA. A: suspensions containing 100 mg/L cyt c and 0 (1) and 200 mg/L (2) HA at pH 7.4 and B: ones containing 70 mg/L hb and 0 (1) and 200 mg/L (2) HA at pH 7.4. (TIF) [file pone.0032818.s004.tif]

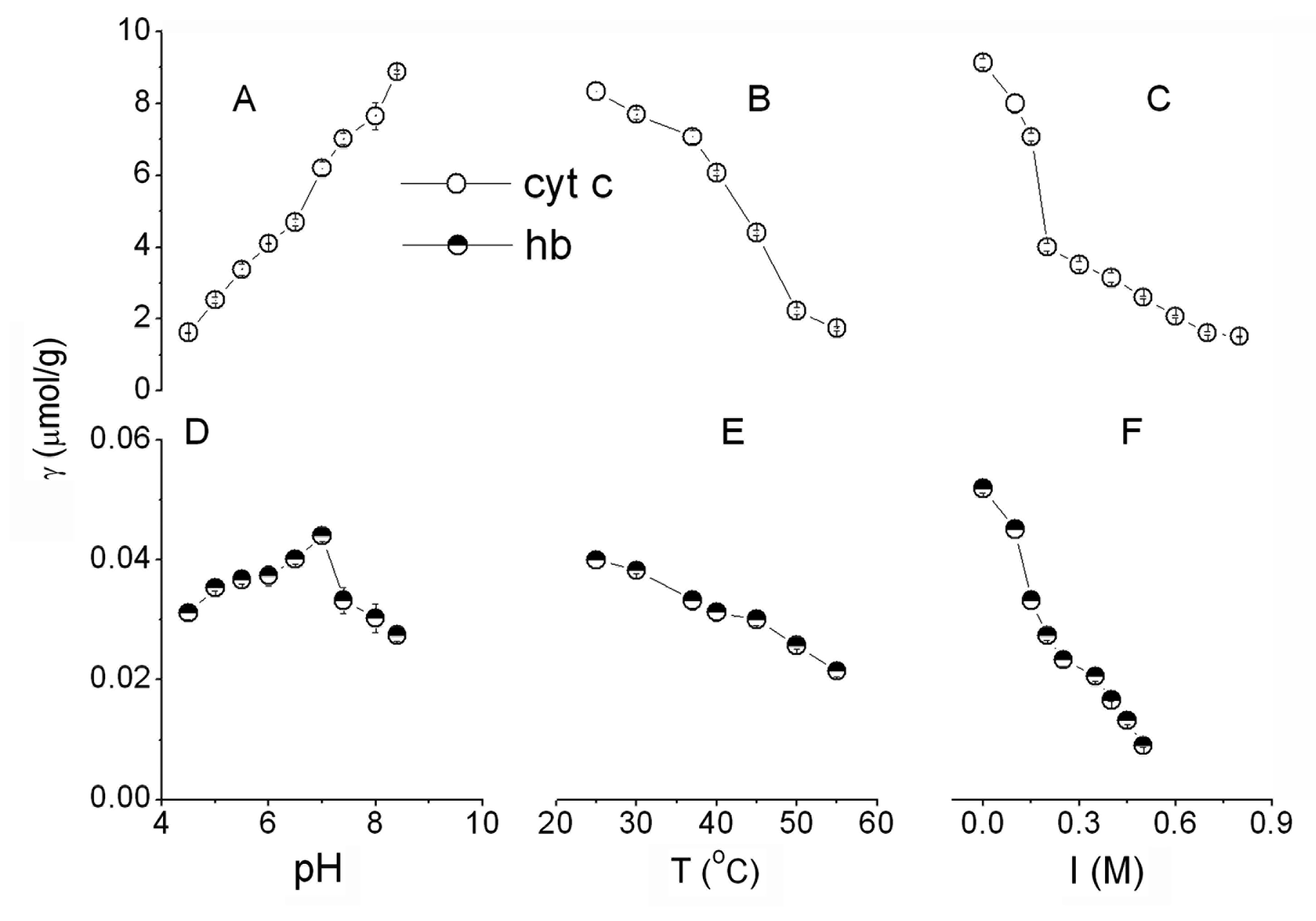

Supplement: Figure S5 — Effects of pH, temperature and electrolyte on γ. pH (A, D), temperature (B, E) and electrolyte (C, F). ○: the cyt c (80 mg/L) - HA (500 mg/L) liquids and ??? : the hb (100 mg/L) - HA (2500 mg/L) liquids. (TIF) [file pone.0032818.s005.tif]

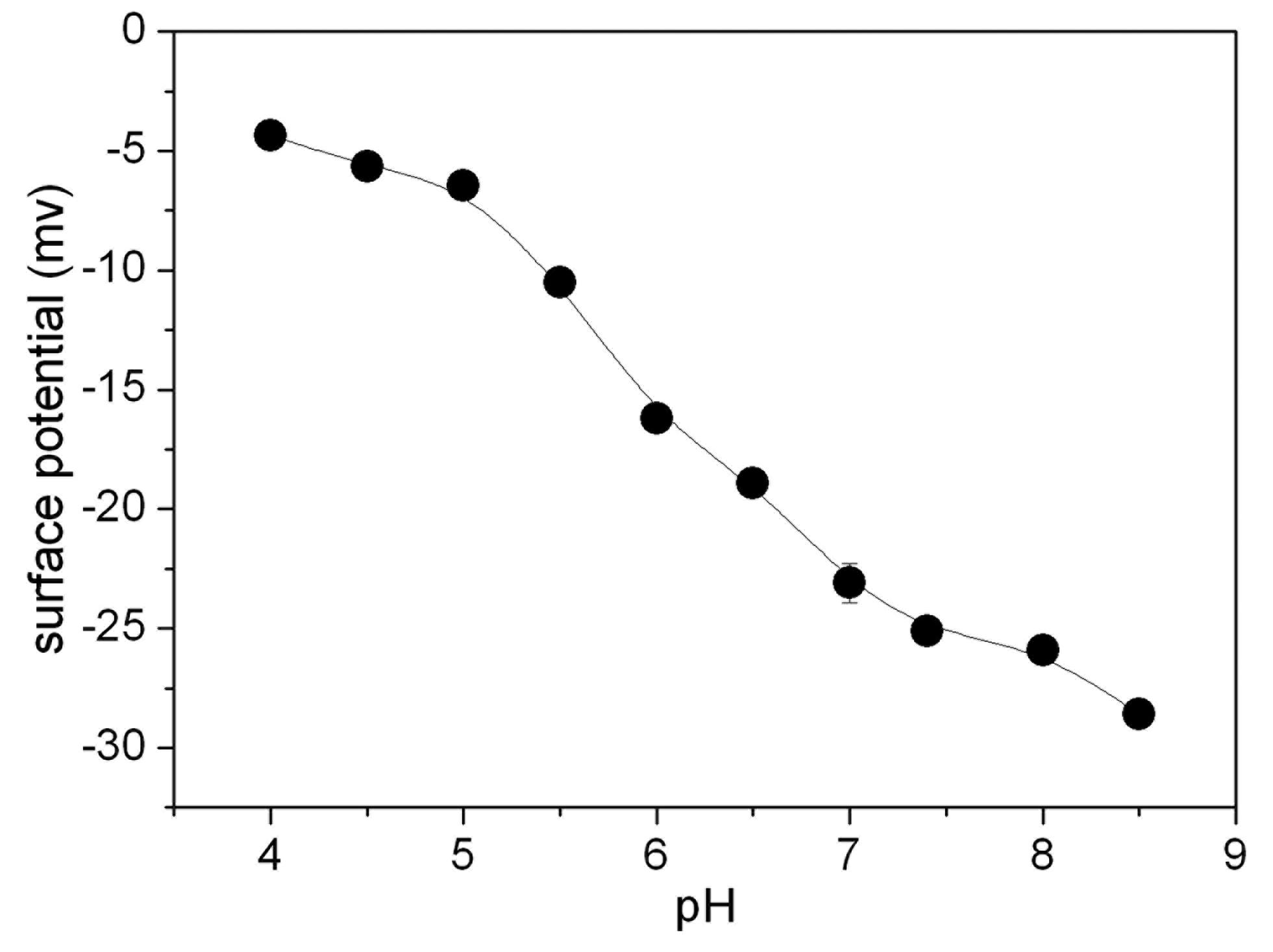

Supplement: Figure S6 — Effect of pH on surface potential of HA. Variation of the ζ - potential of HA with pH. (TIF) [file pone.0032818.s006.tif]

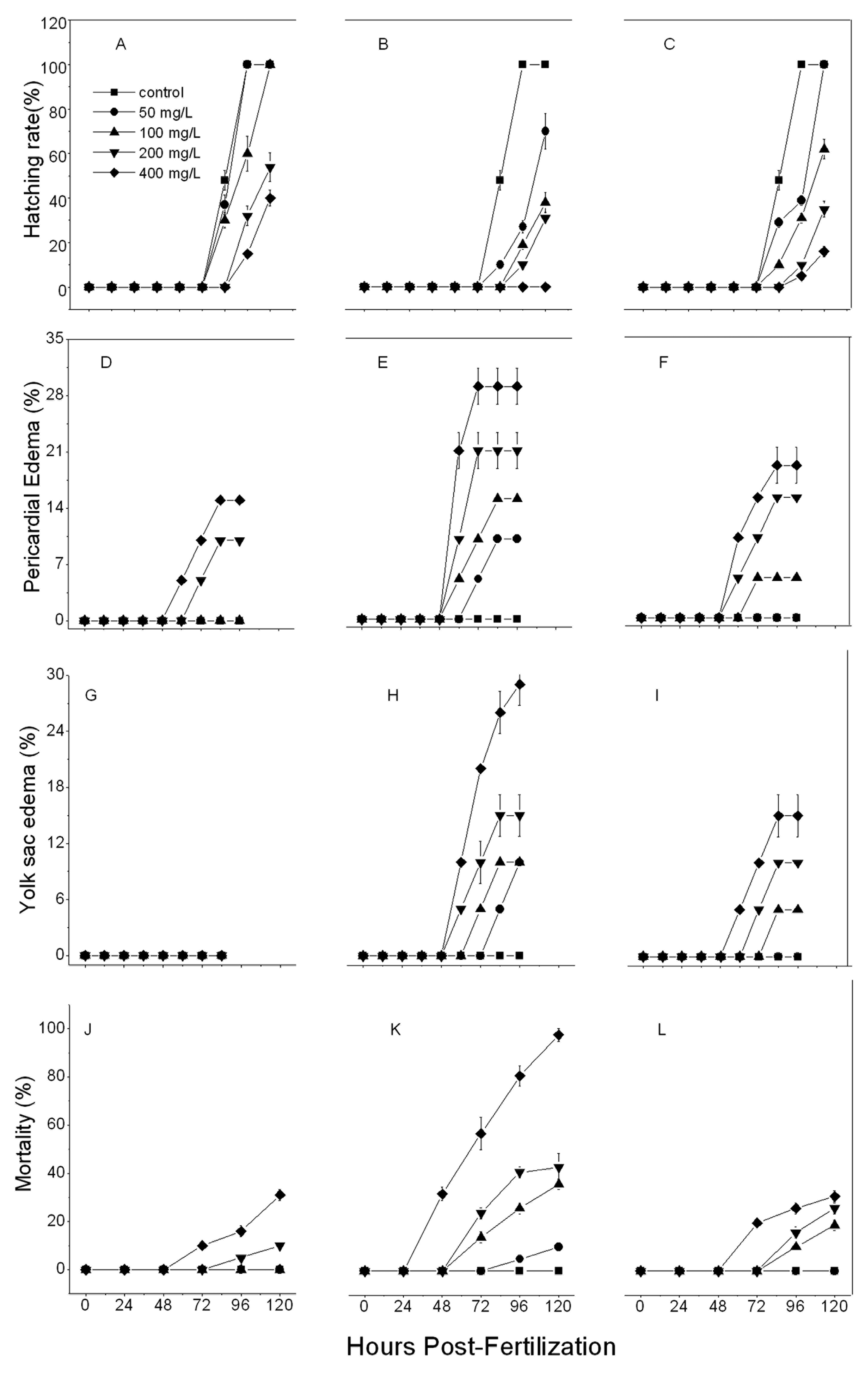

Supplement: Figure S7 — Effects of nanomaterials on hatching rate, pericardial edema, yolk sac edema and mortality. The hatching rate (A to C), ericardial edema (D to F), yolk sac edema (G to I) and mortality (J to L) of zebrafish embryos and larvae exposed in various nano-HA, TiO2 and SiO2 suspensions. (TIF) [file pone.0032818.s007.tif]
